# Formulation considerations in enhancing olfactory mucosal deposition for nose-to-brain drug delivery

**DOI:** 10.1007/s13346-026-02097-7

**Published:** 2026-03-21

**Authors:** Anisha A. DSouza, Maayan Kahan, Alicia Yang, Smrithi Padmakumar, Benjamin S. Bleier, Mansoor M. Amiji

**Affiliations:** 1 Massachusetts Eye and Ear Infirmary, Harvard Medical School, Boston, MA USA; 2 Department of Pharmaceutical Sciences, School of Pharmacy, Bouve College of Health Sciences, Northeastern University, Boston, MA USA; 3 Icahn School of Medicine at Mount Sinai, New York, NY USA; 4 Department of Chemical Engineering, College of Engineering, Northeastern University, Boston, MA USA

**Keywords:** Delivery via olfactory epithelium, Direct nose-to-brain delivery, Olfactory nerve pathway, Trigeminal nerve pathway, Brain targeting, Central nervous system delivery

## Abstract

Nose-to-brain (N2B) drug delivery offers a promising alternative to circumvent the blood–brain barrier and deliver therapeutic agents directly to the central nervous system. Among the intranasal pathways, targeting the olfactory mucosa is particularly attractive due to its direct anatomical and functional connection to the brain. However, effective deposition and retention of drug-loaded formulations in the olfactory region remain significant challenges, owing to complex nasal anatomy, mucociliary clearance, and limited surface area. This review critically examines the physiological and anatomical barriers to olfactory targeting and highlights recent advances in nanoparticle-based strategies designed to enhance mucosal deposition and transport. Various formulation approaches—including mucoadhesive polymers, surface-functionalized nanocarriers, and stimuli-responsive systems—are discussed alongside innovative delivery devices and administration techniques tailored for olfactory mucosal delivery. In vitro, ex vivo, and in vivo models used to evaluate these strategies are reviewed, as are safety, regulatory, and translational considerations. Finally, the review explores emerging technologies such as patient-specific delivery platforms and smart nanoparticles, offering a forward-looking perspective on the future of N2B therapeutics for neurological disorders.

**Graphical abstract:**

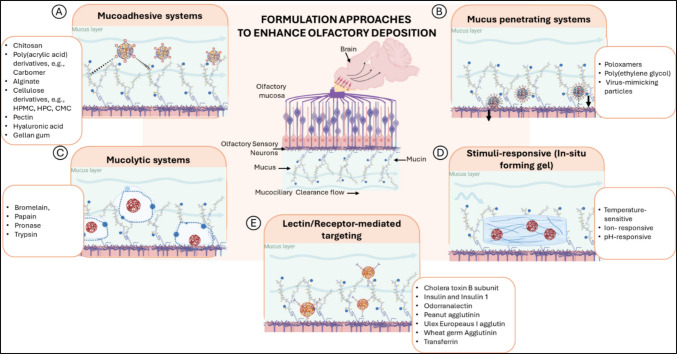

## Introduction

The blood–brain barrier (BBB) is a highly specialized vascular interface that tightly regulates the exchange of molecules between the systemic circulation and the central nervous system (CNS). It is composed of brain microvascular endothelial cells interconnected by continuous tight junctions and supported by surrounding pericytes and astrocytic end-feet, forming a selectively permeable barrier essential for maintaining neuronal homeostasis [1]. While the BBB plays a critical protective role, it severely restricts the entry of many therapeutic agents, particularly hydrophilic compounds and macromolecules, such as peptides, proteins, and antibody-based drugs, into the brain [1, 2]. As a result, pharmacological intervention within the CNS is inherently more challenging than in peripheral tissues, reflecting the brain’s unique immunological and biochemical environment [3].

The BBB, therefore, represents a significant bottleneck in the treatment of neurological disorders. Drugs administered via conventional systemic routes often require high doses to achieve therapeutically relevant brain concentrations, which can lead to increased systemic exposure and a heightened risk of adverse effects. These challenges are particularly concerning in light of the growing global prevalence of CNS disorders associated with aging populations [4]. Although substantial progress has been made in identifying novel therapeutic targets and agents for neurodegenerative diseases such as Alzheimer’s disease and Parkinson’s disease, clinical benefits remain modest, with many treatments demonstrating limited efficacy and poor tolerability, especially in elderly patients [5]. This has intensified interest in alternative delivery strategies that can improve brain targeting while minimizing systemic toxicity.

To address BBB-associated limitations, both invasive and noninvasive approaches have been explored. In oncological applications such as glioblastoma, physical methods including focused ultrasound and laser-based interventions have been investigated to transiently disrupt BBB integrity and enhance local drug penetration [6, 7]. Despite encouraging results, these techniques often require specialized equipment and carry procedural risks, limiting their widespread applicability.

In this context, nose-to-brain (N2B) drug delivery has emerged as a promising noninvasive strategy for CNS targeting. Intranasal administration leverages the anatomical proximity of the nasal cavity to the brain, enabling direct drug transport along neuronal pathways, most notably the olfactory and trigeminal nerves, thereby bypassing the BBB [8]. This route is associated with rapid drug delivery to CNS with reports indicating brain exposure within minutes of administration and widespread cerebral distribution within 30 min, while reducing systemic exposure. Consequently, N2B delivery has gained increasing attention as a versatile platform for improving the treatment of a broad range of neurological disorders.

Drug concentrations detected in brain or CSF following intranasal administration are frequently interpreted as evidence of successful nose-to-brain transport. However, systemic absorption followed by secondary blood–brain barrier passage may substantially contribute to these concentrations, complicating mechanistic interpretation and limiting their validity as definitive markers of direct neuronal delivery [9]. Although N2B administration is non-invasive, its effectiveness critically depends on precise deposition within the olfactory epithelium (OE), the only nasal region with direct anatomical access to the CNS [10]. The OE is therefore the most mechanistically relevant target for true neuronal transport to the brain [10, 11].

Despite its central importance, strategies specifically aimed at optimizing olfactory targeting remain underexplored, with many studies conflating olfactory, trigeminal, and systemic pathways. This review focuses explicitly on formulation and delivery approaches designed to enhance olfactory deposition and examines the key translational challenges that must be addressed to achieve reliable and reproducible nose-to-brain drug delivery.

### Anatomy and physiology of the nasal cavity

The nasal cavity serves as the primary interface between the external environment and the respiratory system, processing millions of inhaled particles with each breath. As the principal organ for olfaction and respiration, the nose is anatomically organized as a paired structure consisting of two symmetric nasal cavities partitioned by a nasal septum. Its intricate anatomy, including the nasal vestibule, septum, turbinate, and specialized mucosal linings, plays a critical role in conditioning inspired air and protecting the host from environmental particulates and pathogens. While this defensive function is highly effective, it also poses substantial challenges for intranasal drug delivery, particularly for N2B applications. The heterogeneous anatomy and regional variability in absorption properties make N2B drug delivery difficult to predict and characterize.

Airflow dynamics of inhaled particles within the nasal cavity are governed by fundamental physical principles, including the Bernoulli effect and Poiseuille flow. They are strongly influenced by individual-specific anatomical features and breathing dynamics. Under normal resting conditions, the primary airflow stream travels through the lower and middle regions of the nasal cavity, mainly between the nasal septum and the middle meatus [12]. Airway resistance varies significantly among individuals and is influenced by factors such as vascular engorgement of the nasal mucosa, septal deviation, turbinate hypertrophy, physical activity, and exposure to irritants [12]. However, another notable contributor to airflow variability is the nasal cycle, also known as the cyclical vascular phenomenon, where the blood vessels innervating the nasal mucosa dilate and constrict in a cyclic manner every three to seven hours [13].

#### Respiratory and olfactory regions

The nasal cavity is broadly divided into two functionally and histologically distinct regions: the respiratory epithelium (RE) and the olfactory epithelium (OE). The respiratory epithelium (RE) in major parts of the nasal cavity, as well as the trachea, bronchi, and has the primary function of humidifying and filtering inspired air. This region is lined with pseudostratified ciliated columnar epithelial cells interspersed with goblet cells, and seromucinous glands, which collectively produce mucus to trap inhaled particles or foreign debris and facilitate their clearance.

In contrast, the olfactory epithelium (OE) is a specialized, thicker, ciliated layer lining the nasal roof, including the olfactory cleft and superior-posterior septum, superior turbinate, and superior and medial aspect of the middle turbinate [12]. The OE contains sensory neurons, supporting cells, and basal cells for olfaction and represents the only direct communication between the external environment and the brain [8, 14]. The human olfactory system consists of approximately 6 to 30 million bipolar receptor cells, although this number decreases with age [15]. During normal respiration, the olfactory mucosa is sheltered, limiting olfaction with limited airflow [12]; however, sniffing redirects airflow towards OE, enhancing odorant and possibly drug delivery.

**Fig. 1:**
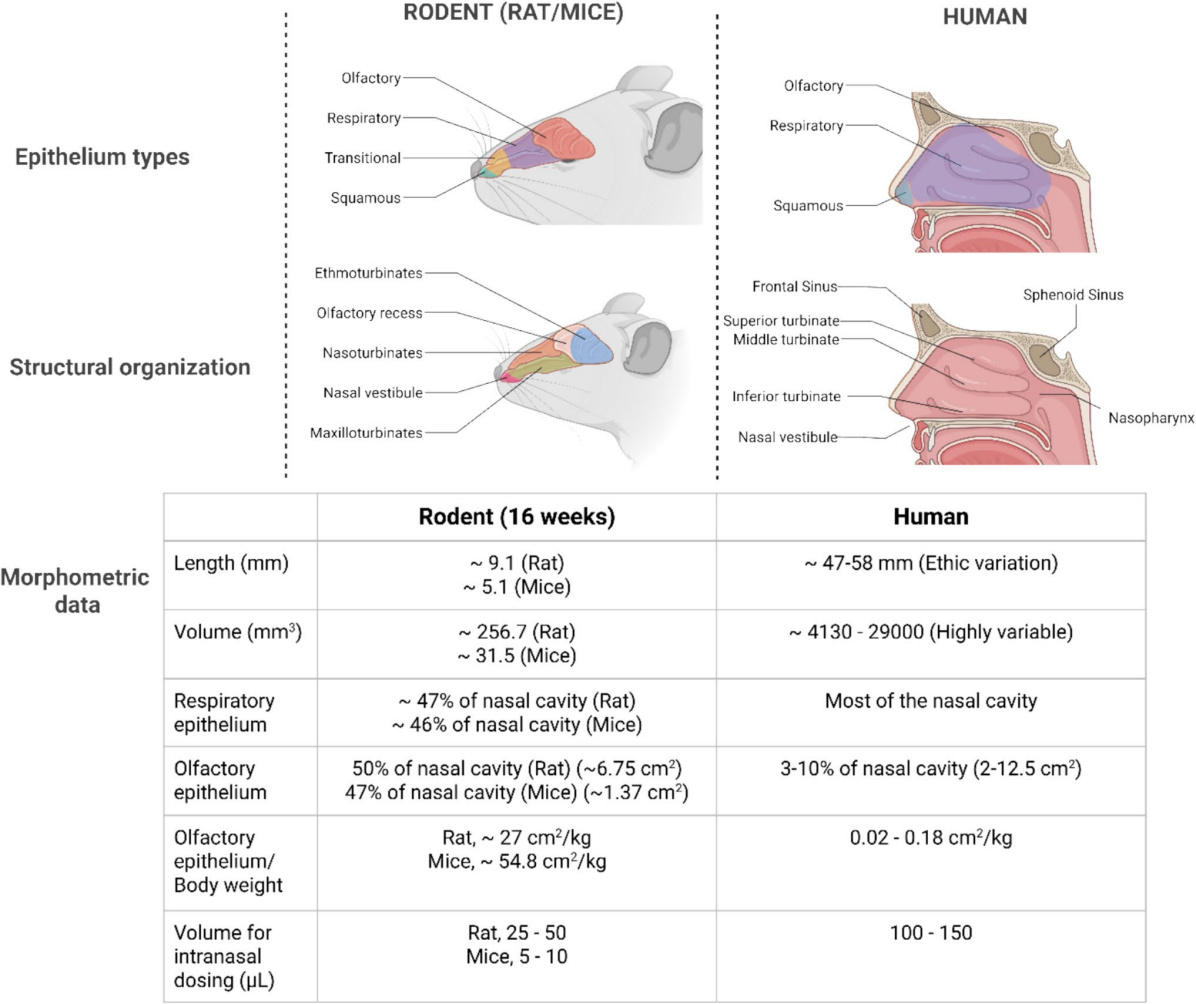
Cross-species comparison of epithelial organization, nasal cavity architecture, and morphometric parameters in adult rodent and human nasal cavities. Schematic illustration depicting the distribution of respiratory, olfactory, and transitional epithelia within the nasal cavities of adult rodents and humans, together with species-specific differences in structural organization. Created in BioRender https://BioRender.com/owyocdg. The table in the lower panel incorporates the key morphometric parameters relevant to N2B delivery. Data synthesized from published literature [21–27]

Anatomical differences between species present additional challenges in translating N2B drug delivery systems from preclinical models to humans. Olfaction is significantly more important for rodents and canines than for monkeys and humans, resulting in evolutionary differences in anatomy and physiology. In humans and monkeys, the nasal vestibule is larger, and the turbinates are simpler than in rodents (Fig. 1) [16]. Although the overall surface area of the human nasal cavity is greater, the proportion of OE is significantly smaller in humans compared to commonly used animal models [17]. These interspecies differences substantially affect airflow patterns, particle deposition, and drug targeting efficiency, complicating direct translation of preclinical findings [18–20].

#### Mucus – cilia interactions

The entire nasal cavity is lined by mucosa; however, distinct anatomical regions differ markedly in composition, vascularization, absorption capacity, and neural connectivity, all of which influence drug deposition and transport pathways. For N2B delivery, the drug should reach the peripheral nerve endings of either olfactory sensory neurons (OSN) or those of the trigeminal nerve, thereby enabling direct transport to reach the brain and CNS. The anatomical connectivity between these neuronal pathways and the brain is discussed in detail in "Rationale for targeting the olfactory mucosa".

In humans, conventionally administered nasal formulations predominantly deposit in the anterior nasal cavity, i.e., the vestibule and RE. These regions are therefore commonly exploited for local delivery of drugs such as local anesthetics, glucocorticoids, or decongestants for local administration [28]. The RE, occupying almost 90% of the human nasal cavity surface area, is covered with respiratory mucosa [29]. Nearly 80% of the epithelial cells in this region bear more than 100 motile cilia per cell [30], which play a major role in mucociliary clearance.

Mucus in the RE mucosa is secreted by seromucous glands, goblet cells, and basal cells and is rich in mucins. The relative abundance of individual mucins varies under pathological conditions, with MUC5B being more strongly associated with airway defense than MUC5AC [31]. In addition to mucins, the nasal mucus contains numerous antimicrobial proteins, contributing to the innate immune defense [32]. Consequently, exogenous materials, including intranasally administered drugs, are cleared rapidly from the respiratory mucosa. Structurally, the mucus layer is of two layers: a low viscosity periciliary layer adjacent to the epithelial cells and a more viscous, gel-like layer overlaying it [33]. The tip of the ciliated cells can propel only the mucus in the periciliary layer. Too slippery mucus just drips out of the nose or into the lungs, and too viscous and sticky can never be propelled [33, 34]. Coordinated beating of respiratory ciliated cells drives mucus towards the nasopharynx, where it is swallowed and digested [35].

**Fig. 2:**
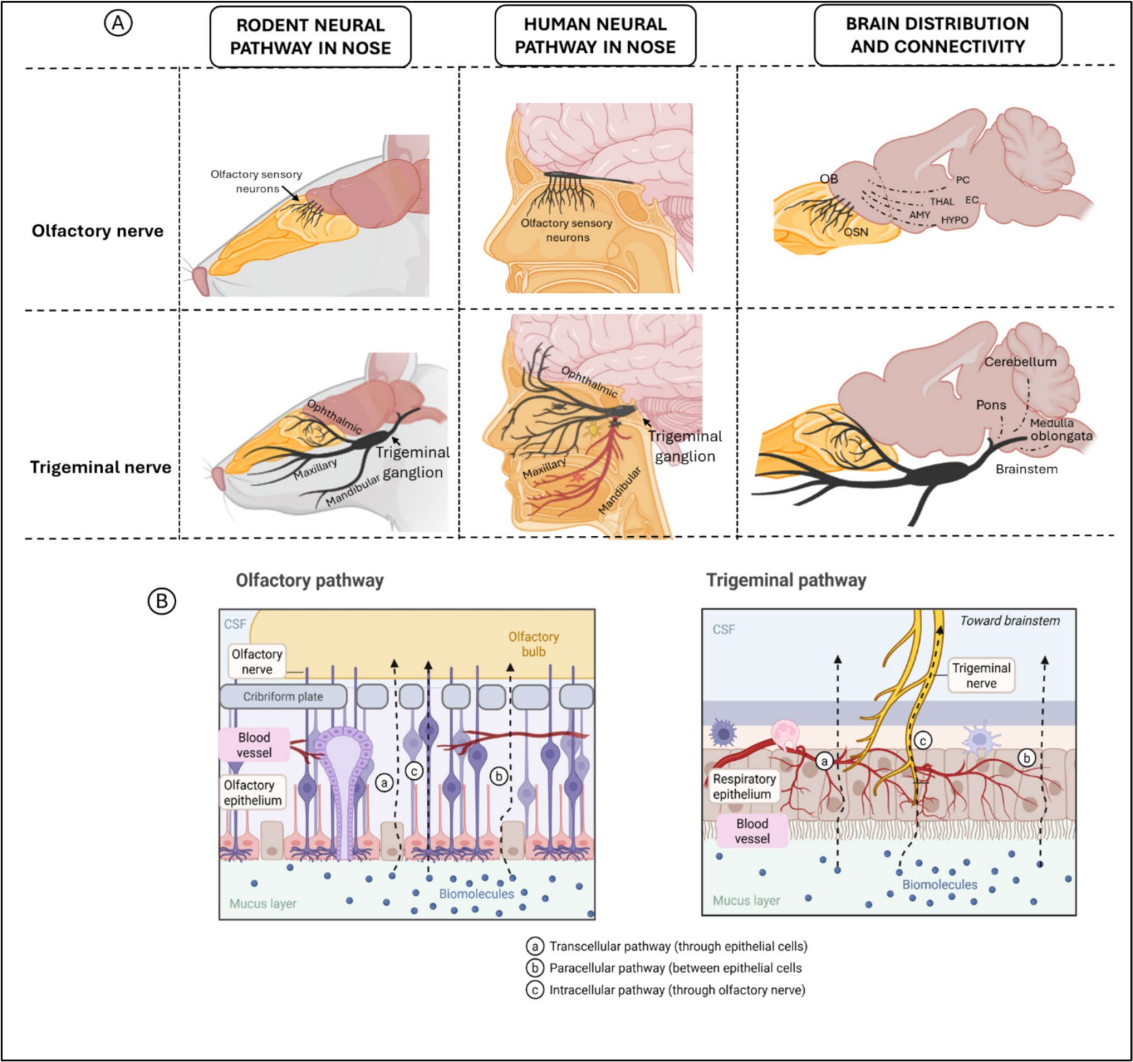
Cross-species of olfactory and trigeminal neural pathways and transport mechanisms in the nasal cavities mediating direct nose-to-brain connectivity. (**A**) Schematic illustration depicting the distribution of olfactory sensory neurons (OSNs) and trigeminal nerve branches within the nasal cavities of adult rodents and humans. OSNs originating in the olfactory epithelium project to OB—olfactory bulb, with downstream connectivity to AMY—Amygdala, EC—Entorhinal cortex, HYPO—Hypothalamus, PC—Piriform cortex, THAL—Thalamus. In parallel, trigeminal nerve branches innervating the mandibular, maxillary, and ophthalmic regions provide an additional direct conduit to the brainstem, pons, cerebellum, and Medulla oblongata. In humans, OSNs traverse the OE, while trigeminal traverse RE. (**B**) Substances may enter perineural spaces en route to the OB, brain stem, or cerebrospinal fluid via transcellular (a), paracellular (b), or intracellular/intraneuronal pathway (c). The RE also increases the potential of systemic absorption. Created in BioRender and MS PowerPoint. https://BioRender.com/7w33rij; and modified from [40]

The trigeminal nerve endings implicated in N2B delivery are located beneath the tight junctions of the apical epithelial cells and do not directly penetrate through the surface of RE, remaining a few microns below the interface (Fig. 2). Moreover, the RE is highly vascularized, with a larger relative density of blood vessels than the OE. As a result, drugs deposited in this region are more likely to undergo systemic absorption rather than neural transport to the brain [36] as seen with calcitonin, desmopressin, and sumatriptan [28].

In contrast, the olfactory mucosa provides direct exposure of the apical dendrites of OSN to the external environment or inhaled air. OSNs extend non-motile primary cilia into the mucus layer, potentially increasing interaction with therapeutics (Fig. 2). The trigeminal nerve endings, primarily from the ophthalmic branch, are also present [37] but they terminate just beneath the epithelial tight junctions [37].

Given the direct exposure of OSNs to pathogens and environmental insults, OSNs survive for approximately one month. Regeneration is mediated by horizontal basal stem cells, while Bowman’s gland supports mucus secretion. This differs from RE mucosa, where mucus production relies on goblet cells mainly [28].

Although the absence of motile cilia on OSNs might suggest reduced mucociliary clearance in OE, several mechanisms may compensate for this limitation. These include gravitational drainage of mucus towards RE, or continuous secretion from Bowman’s glands, or maybe small islands of RE allowing ciliated cells to contribute to mucociliary clearance. There is no distinct boundary between OE and RE; OE exists as patches within the RE [38, 39]. MUC5AC is the predominant mucin in the OE [31] also implies lesser implication for pathogen clearance and high drug residence within OE.

### Rationale for targeting the olfactory mucosa

In humans, conventionally administered nasal drugs tend to deposit primarily in the anterior nasal cavity, i.e., the RE. The RE is highly vascularized, with a larger relative density of blood vessels than the OE; hence drugs can be picked up by the blood instead being directly delivered to the brain [36]. Tmaking up about 3–10% of nasal surface area [41, 42]. Ifunctional.

mitral and tufted cells of[36, 42]project to the piriform cortex, hypothalamus, anterior olfactory nucleus, and amygdala [36]. [40, 44].

On the other hand, the trigeminal nerve present in the RE consists of three branches—ophthalmic (V1), maxillary (V2), and mandibular (V3). However, only the ophthalmic and maxillary branches, and not the mandibular branch, are involved in N2B delivery [45]. Unlike the olfactory nerve, the trigeminal nerve enters the brain at two distinct locations—near the pons and second—the olfactory bulb through the cribriform plate. This enables the trigeminal nerve pathways to access both the caudal and rostral regions of the brain [36]. However, the trigeminal nerve is significantly longer than the olfactory nerve, stretching 20 mm compared to the olfactory nerve’s 4 mm [42], which may limit transport efficiency.

In summary, in the OE, besides the OSN, the innervation by the ophthalmic branch of the trigeminal nerve provides an additional intraneural pathway to the brain stem, thalamus, and limbic system [12]. Drugs transported along the trigeminal pathways can reach the cerebellum and pons, and subsequently distribute throughout the brain via both intracellular and extracellular mechanisms [44].

The anatomical and functional differences between the OE and RE are therefore critical considerations in the design of N2B drug delivery systems [40]. Key factors influencing delivery efficiency include nasal anatomical constraints, physicochemical properties of the drug affecting CNS bioavailability, carrier systems that modulate transport and target interaction, and delivery techniques capable of preferentially depositing formulations within the olfactory region [40]. Although the OE presents significant promise as a target for N2B delivery, multiple biological and anatomical barriers limit the full realization of its therapeutic potential.

## Mechanisms of nose-to-brain transport

Discriminating the dominant transport mechanisms involved in N2B delivery remains challenging, as multiple pathways may operate simultaneously depending on formulation properties, particle size, and physiological conditions.

### Intraneuronal Transport

Intraneuronal transport refers to the direct endocytic uptake of molecules by neurons, followed by slow axonal transport (Fig. 2) [41, 46]. Cellular internalization can occur through either the nonspecific endocytosis mechanism of pinocytosis or the receptor-mediated endocytosis mechanism of phagocytosis. Direct uptake at the receptor neuron is both one of the slowest, with a travel rate ranging from 25 to 130 mm/day, but it is the most anatomically direct pathway for N2B [42, 46]. Receptor-mediated transport is particularly advantageous in transporting large molecules and ligands that exhibit affinity for the neuronal surface receptors [36].

The OSN is one of the smallest axons in the CNS, with the axonal diameter ranging between 0.1 and 0.7 μm [46]. Following axonal transfer, the internalized molecule or drug is directly released into the postsynaptic neurons within the olfactory bulb through exocytosis, bypassing both the BBB and barriers of lamina propria [41]. Exocytosis may occur through synaptic, extrasynaptic, or dendritic vesicular pathways [41]. Wheat-germ agglutinin horseradish peroxidase has demonstrated receptor-mediated endocytosis, in both olfactory neurons and the trigeminal nerve [44]. In contrast, nanogold-labeled insulin undergoes receptor-mediated internalization selectively in the olfactory nerve, but not in the trigeminal nerve [44]. Bacilli like *Burkholderia pseudomallei* can be taken up directly via the trigeminal nerve and transported directly to the brain stem and spinal cord within 24 h; however the underlying mechanism for this remains poorly understood [47].

### Extraneuronal transport

Extraneuronal pathways facilitate N2B delivery through paracellular or transcellular diffusion across the tight junctions of nasal epithelium, followed by perineural transport through the lamina propria to the brain, again circumventing the BBB [41, 42, 48]. The cerebrospinal fluid (CSF) further supports intercompartmental transport, enabling solute movement between the nasal cavity and intracranial spaces [44]. This process is notably faster than intraneuronal transport and may be particularly relevant for small, hydrophilic molecules and nanoparticulate systems [46].

The nasal epithelium exhibits both charge-selective and size-selective permeability pathways. Size-selective paracellular pathways permit particles through pores with estimated diameters between 30 and 60 Å, whereas the charge-selective pathway is limited only to particles with diameters between 4 and 8 Å [41]. In addition, the dynamic turnover of an OSN is typically between 30 and 60 days [44], resulting in continuous epithelial remodeling. The presence of actively proliferating and differentiating cells also provides gaps in the epithelium for the particle to traverse [42].

Transcellular transport across the epithelial cells may also occur via receptor-mediated endocytosis involving receptors such as the insulin receptor and the transferrin receptor [49].

### Transport of inorganic nanoparticles

Inorganic nanoparticles such as silica [50], gold [51], quantum dots [52], iron or metal oxides [53] offer high drug-loading capacity and can be readily functionalized to enhance brain targeting. Following intranasal administration, these nanoparticles can also access the brain through olfactory and trigeminal neuronal pathways, as well as via indirect lymphatic and vascular routes [54]. Their small size facilitates widespread distribution within brain tissue [55]. In addition, certain inorganic nanocarriers can be engineered as stimulus-responsive or multifunctional platforms, enabling activation by external triggers such as ultrasound [56, 57] or magnetic fields [53, 58] or designed for multimodal applications [59, 60]. For example, aerosolized quantum dots (~ 15 nm) have demonstrated rapid uptake via olfactory axonal transport [52]. MicroCT analysis studies further confirmed that β-cyclodextrin–chitosan coated gold–iron oxide nanoparticles (~ 50 nm) can migrate along olfactory and trigeminal nerves, with additional paracellular transport across the olfactory epithelium [59]. Despite these advantages, concerns remain regarding potential toxicity, limited drug release, and incomplete systemic clearance of inorganic nanoparticles [61–63]. In view of these limitations and the substantial investigation required to address safety and clearance concerns associated with inorganic systems, this review concentrates primarily on conventional organic nanoparticle platforms.

### Bidirectional neural transport and clearance

Neuronal transport is inherently bidirectional, involving both anterograde and retrograde movement, which may have implication for N2B delivery. Although therapeutic agents may access the brain via the olfactory and trigeminal pathways, these same routes may also facilitate redistribution or efflux, thereby contributing to clearance ‘tug-of-war’ model [64]. Retrograde axonal transport in olfactory neurons has been demonstrated using wheat germ agglutinin and horseradish peroxidase, as well as neurotropic viruses [65, 66].

Additionally, CSF dynamics also play a central role in CNS homeostasis and solute clearance. A proportion of CSF drains through perineural pathways across the cribriform plate into nasal and cervical lymphatics, forming part of the glymphatic-lymphatic continuum [67, 68]. Drug uptake through the OE must therefore occur against ongoing lymphatic drainage [69]. Notably, however, this route accounts for a relatively small fraction (typically < 5%) of total CSF outflow [68, 70, 71]. CSF absorption and turbinates-associated lymphatics outflow resistance are further influenced by environmental conditions and intranasal interventions, which may influence the lymphatic contractile activity [68, 72].

Preclinical studies indicate that after an intranasal administration, initial CNS uptake is rapid, particularly within the first 30 min after dosing, whereas elimination proceeds more gradually. Systemic redistribution via CSF circulation and peripheral metabolism appears to be the dominant clearance mechanism. Nevertheless, certain tracers including Texas Red–labeled dextran, Texas Red-labelled Dextran, ^86^Rubidium, ^201^Thallium demonstrated measurable efflux through olfactory-associated pathways [73]. Despite these findings, robust evidence in humans remains limited. [74]. For example, MRI studies following intrathecal gadobutrol administration revealed tracer accumulation near the cribriform region but not within the nasal mucosa or turbinates after 48 h. This suggests that under physiological conditions, nasal CSF clearance may be limited [75]. Furthermore, pathological states such as tumors, stroke, or neurodegenerative disorders can further disrupt CSF dynamics and glymphatic function, potentially reducing CNS outflow [68].

Viral systems exploiting trigeminal retrograde transport have also been investigated therapeutically for trigeminal neuralgia [76] and other disorders affecting motor neurons [77]. In the former study, retention within trigeminal persisted for several hours. However, the brain levels were found to be sustained and higher compared to oral administration. The absorption kinetics along the trigeminal pathway were slower than those of the olfactory route [76], further highlighting pathway-specific differences in transport dynamics. Pegylated lipid nanoparticles containing ionizable amino lipid and FAM-tagged RNA showed dissociation of RNA from LNP and demonstrated a retrograde transport towards soma of 2D culture of cortical neurons [78].

Finally, the CNS dynamics itself is pharmacologically modifiable. Agents such as acetazolamide [79] and methotrexate [80] reduce CSF production and have been associated with increased brain drug concentrations, whereas prostaglandin analogues enhance CSF outflow [81]. These observations suggest that CSF physiology could represent a secondary determinant of drug retention and elimination following nose-to-brain delivery.

## Olfactory mucosal delivery challenges

Despite the numerous advantages, N2B delivery is significantly hindered by anatomical, physiological, and patient-related factors. These barriers collectively impede targeted deposition within the OE and diminish both reproducibility and predictability of therapeutic outcomes, and hence the translational potential of intranasal drug delivery systems [82, 83]. Understanding these constraints is essential for rational design of intranasal formulations optimized for CNS delivery.

### Anatomical constraints

The human nasal cavity has an overall surface area of ~ 150 cm^2^ and a total volume of ~ 15 mL [44, 84]. In addition to this geometrically intricate environment, the labyrinthine architecture, coupled with tortuous airflow dynamics, results in reduced velocity and recirculatory flow beneath the OE region, leading to suboptimal deposition within the OE [85].

The spatial organization of nasal epithelia becomes even more complicated. The vestibular region, encompassing an overall surface area of 0.6 cm^2^, exhibits limited drug absorption due to poor permeability. The RE encompasses approximately 130 cm^2^, exhibits dense vascularization, and has extensive microvilli, promoting rapid systemic absorption instead of permitting N2B delivery [86]. In contrast, the OE constitutes only 2–12.5 cm^2^ (~ 10% of the human nasal cavity), positioned in the superior-posterior cavity, which limits access and deposition [86, 87]. The OE’s specialized architecture, including olfactory sensory neurons (OSNs) and trigeminal nerve branches, provides unique intraneural conduits to the CNS [88, 89]. However, its restricted area highlights the need for delivery strategies that can target regions precisely. Moreover, the limited volume of just 100–150 µL administration per human nostril does not permit the molecules to reach the OE efficiently, hindering the efficacy of CNS-active drugs [86]. Notably, interspecies variations in OE surface area and structural arrangement pose additional translational challenges when extrapolating preclinical findings to human applications [87].

### Physiological Barriers – mucus and mucociliary clearance

In addition to anatomical constraints, nasal mucus and mucociliary clearance are significant physiological barriers that limit the intranasal residence time of administered therapeutics. Mucus is cleared at intervals of 10–15 min, with a total daily secretion of 20 to 40 mL, substantially restricting epithelial contact time [28, 90, 91].

Upon secretion, the gel-forming mucins rapidly expand in the biological milieu through ionic interactions with cationic charge present there, resulting in an approximately 500-fold increase in mucus gel volume [31]. This entire process occurs within 50 ms, after which it is propelled from the anterior nasal cavity towards the oropharynx by the coordinated ciliary beating activity in the RE, thereby protecting the mucosa from exogenous substances [88, 92].

Formulation strategies, such as mucoadhesive agents or nanoparticle-based systems, may improve epithelial interaction, prolong the total nasal residence time, and thereby increase drug absorption [93, 94]. However, their effect may vary across nasal regions. The OSNs in the OE possess non-motile primary cilia and, therefore, do not significantly participate in the mucociliary clearance [95], which could potentially prolong drug exposure.

Beyond bulk clearance, membrane-bound mucins introduce an additional dynamic barrier. The mucins contain SEA domains comprising enterokinase, arginine, and sea-urchin sperm proteins, capable of auto-proteolysis, leading to ‘mucin shedding’ and continuous remodeling of the mucus layer [28]. Furthermore, nasal physiology is influenced by the nasal cycle, characterized by alternating airflow dominance between nostrils of approximately 50 min to 4 h, [96], which further enhances the variability in drug deposition and clearance.

### Physiological barriers – enzymes and transporters

Beyond structural limitations, dynamic nasal physiology imposes substantial constraints on drug residence and absorption. A volume of 12,000 L of air passes through the nose and is modulated by a 0.3–0.6 cm^2^-sized nasal valve [97]. The nasal valve and vestibule regions contribute to 52.6–78.3% of the total airway resistance of the nasal cavity [98] generating the highest airflow resistances in the narrowest segment, i.e., in the superior cavity of OE, and higher airflow velocities in anterior regions [97, 99]. The triangular shape and the dynamic narrowing of the nasal valve are the bottlenecks for N2B delivery.

The presence of degrading enzymes and efflux transporters also presents significant challenges to N2B delivery. Hydrolytic enzymes, such as cytochrome P450 isoenzymes, carboxylesterases, epoxide hydrolases, carbonic anhydrase, aminopeptidases, proteases, glutathione S-transferase, aldehyde dehydrogenase, and UDP-glucuronyl transferase, can metabolize drugs and xenobiotic substances in the nasal lumen or in the epithelial layer before CNS uptake [100]. The olfactory submucosa also harbors efflux transporters, such as P-glycoprotein and Multidrug Resistance Protein 1 (MRP1). Efflux transporters prevent the influx of drugs, thereby reducing their absorption into the nasal membrane [101–103].

### Patient-specific variability

Inter-individual heterogeneity can markedly affect therapeutic absorption following intranasal administration. Variations in nasal physiology, mucosal condition, and anatomical architecture may significantly influence regional drug deposition, absorption dynamics, and therapeutic reproducibility. Conditions such as infections, rhinitis, allergies, pre-existing illnesses, or pathological conditions, can alter the nasal pH, mucosal production, integrity, clearance, and interactions, thereby affecting the rate of absorption and drug transport dynamics [42, 101]. Anatomical variations, including septal deviation, turbinate hypertrophy, or differences in nasal cavity volume, as well as gender, ethnic, and age-specific differences, can contribute to additional variability in therapeutic outcomes [104–107]. Host-specific factors therefore introduce variability in N2B delivery and therapeutic efficiency. This highlights the need for adaptable device design, optimized administration techniques, to ensure consistent and effective therapeutic outcomes in N2B development [91, 108, 109].

## Strategies to enhance olfactory mucosal deposition

Efficient delivery of therapeutics via the olfactory pathway requires precise deposition within the upper or superior nasal cavity, particularly the OE. Following deposition, drug molecules must traverse the nasal mucus layer and epithelial barrier to reach the CNS through olfactory neurons and associated perineural pathways. Achieving consistent and efficient olfactory deposition is challenged by anatomical constraints, physiological clearance mechanisms, and drug-related limitations explicitly covered in "Olfactory mucosal delivery challenges".

**Fig. 3:**
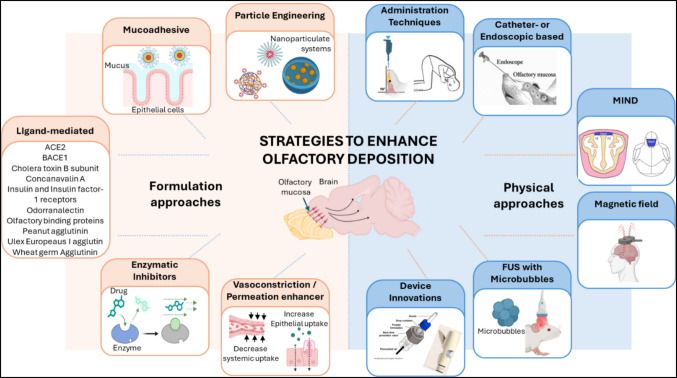
Overview of nose-to-brain drug delivery strategies. Broadly, the strategies can be categorized as non-invasive or formulation-based approaches and invasive approaches. The non-invasive approaches include particle-engineered nanoparticulate systems, muco-adhesive systems, ligand-mediated delivery, vasoconstrictors, and enzyme inhibitors. Invasive approaches involve surgical interventions or physical modalities such as monitoring the position of the rodent or human body, catheter-assisted and endoscopic-based delivery, Minimally Invasive Nasal Depot showing the coronal anatomical view and orientation of the drug depot in the rodent snout, externally applied magnetic fields, Focused ultrasound with microbubbles, and Specialized intranasal delivery devices. The human posture shown in the inset is adapted from [114, 115] and is licensed under Creative Commons CC-BY-NC. The endoscopic image is reproduced from [116] under Creative Commons Attribution (CC BY) license. MIND images are adapted from [117]. Additional schematic elements are created using BioRender. https://BioRender.com/bzyx163

N2B delivery strategies can be broadly classified into invasive or non-invasive approaches [110]. Non-invasive strategies include nano-formulations, muco-adhesive systems, ligand-mediated delivery, the use of vasoconstrictors, enzyme inhibitors, and penetration enhancers to enhance epithelium transport (Fig. 3). Invasive approaches entail surgical interventions or physical modalities such as catheter-assisted and endoscopic-based delivery, Minimally Invasive Nasal Depot, Specialized intranasal delivery devices, using magnetic fields, and focused ultrasound with microbubbles. Despite significant advances, N2B formulations require further optimization to enhance targeting precision, minimize systemic exposure, and achieve a rapid onset of CNS action [111].

Reports of permeability differences between RE and OE further complicate N2B delivery. Studies using animal models, such as sheep, have demonstrated variability in compound permeation between the nasal concha (RE) and the ethmoid concha (OE) [112, 113]. While compounds such as rhodamine 123, Lucifer Yellow, FITC-dextran, and caffeine exhibited higher permeation across the OE, these differences were not consistently statistically significant for all molecules, as exemplified by atenolol. These findings underscore the compound-dependent nature of olfactory transport and highlight the need for tailored strategies that enhance deposition.

### Formulation Approaches

**Fig. 4:**
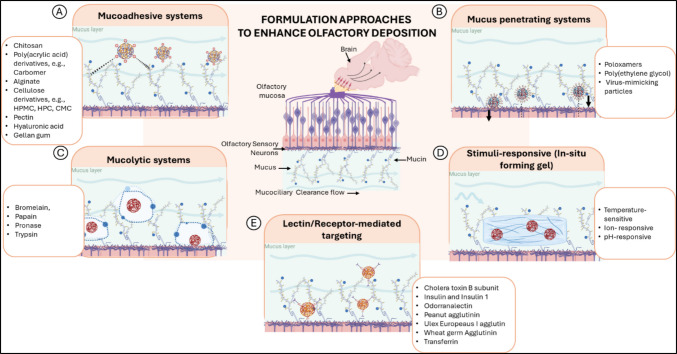
Possible Formulation approaches for Nose-to-Brain delivery through the Olfactory mucosa. (**A**) Mucoadhesive-containing formulations interact with the hydrophobic and hydrophilic glycan segments of mucin. (**B**) Mucus-penetrating formulations penetrate through the mucus gel so that they can reach the epithelium. (**C**) Mucolytic enzymes immobilized on particles cleave mucin glycoproteins through amide bond hydrolysis and increase mucus permeability. (**D**) Stimuli-responsive hydrogels undergo in situ sol–gel transitions, thereby prolonging nasal residence. (**E**) Lectin- and receptor-mediated targeting exploits specific interactions with surface molecules on the OE. Created in BioRender and MS PowerPoint

Mucoadhesive and bioadhesive polymers, such as chitosan, poly(acrylic acid), hyaluronic acid, and gellan gum, interact with mucin glycoproteins to enhance formulation-mucosa contact, prolong nasal residence time, and improve drug absorption [110, 118]. These interactions are facilitated by the heterogeneous structure of mucin fibers, which contain both hydrophobic domains associated with cysteine-rich regions and hydrophilic regions resulting from the negative charge of the glycan segments [28]. As a result, mucins can interact with both hydrophilic and hydrophobic particulate systems (Fig. 4A). Additionally, the negatively charged proteoglycans on the mucosal surface further provide opportunities for targeting cationic polymers/lipids through weak and reversible electrostatic interactions. Such charged interactions are dependent on pH and ionic strength [119].

Mucopenetrating excipients, such as poly (ethylene glycol) and poloxamers, do not interact with the biological milieu, ensuring minimal interaction between the formulation and mucus (Fig. 4B) [120]. Particle size of the formulation is also an important determinant of mucus penetration. Smaller-sized particles enhance diffusivity and are better able to navigate the mucin mesh, which is characterized by pore sizes ranging from approximately 20 to 200 nm [121]. Designing delivery systems that balance mucus penetration with sufficient epithelial adhesion should be considered for intranasal drug delivery. Another strategy to reduce the mucus barrier involves immobilizing mucolytic enzymes, such as papain, bromelain, trypsin, and pronase. These enzymes cleave mucin glycoproteins through amide bond hydrolysis, thereby increasing mucus permeability and enhancing formulation diffusivity (Fig. 4C) [120].

Chitosan, in particular, transiently opens tight junctions and improves brain bioavailability of peptides and small molecules. A 0.5% chitosan solution containing insulin (pH 4.0) increased brain bioavailability by 15% relative to subcutaneous administration and 7% compared with intravenous delivery in sheep [118]. Likewise, clomipramine-loaded chitosan aerogels (13–59 µm) achieved rapid distribution to the frontal cortex and hippocampus within 30 min post-intranasal administration [122]. Chitosan, however, exhibits mucoadhesive properties within a specific pH range and becomes increasingly soluble under acidic conditions [123]. This pH-dependent solubility may present challenges for the delivery of drugs that are unstable in acidic environments. Furthermore, chemically modified chitosan derivatives have generally demonstrated enhanced mucoadhesive performance compared with native chitosan, owing to improved interaction with mucosal surface. Dry powders are generally preferred over solutions as they can be retained more effectively on the mucosal surface [124, 125].

Although, mucoadhesive polymers have been widely investigated for intranasal delivery, their specific effects or interactions with the OE remain underexplored and warrant further investigation. Notably, the OSN on OE has non-motile cilia, and therefore do not contribute to mucociliary clearance [95]. While mucoadhesion may prolong residence time, effective formulations must also penetrate through the mucus layer and adhere to the epithelial surface. The nasal mucosa is characterized by rapid mucociliary clearance (intervals of 10–15 min) and continuous mucus turnover, which may clear off the formulations. Mucus is a highly viscoelastic, multilayered barrier that is difficult to immobilize; its thickness and viscosity may increase under pathological conditions. Although the thickness of the mucosal layer is not clearly defined in OE region, the respiratory tract has been reported to be approximately 10 μm [126]. Additionally, formulations must continuously diffuse against continuous mucus flow directed towards the nasopharynx [34].

Stimuli-responsive hydrogels are promising systems for intranasal and N2B delivery due to their ability to undergo in situ sol–gel transitions, which prolong nasal residence and enhance drug bioavailability (Fig. 4D). These formulations remain inactive under normal conditions and release the drug only in response to specific stimuli, enabling controlled delivery. Several reviews have discussed their design and applications [124, 127, 128]. Further details are provided in "Smart and stimuli-responsive delivery systems".

### Delivery device innovations

Optimized device-assisted intranasal delivery is essential for translating N2B delivery into clinical practice. However, its success is strongly dictated by the integration of formulation design with delivery device performance [95]. Conventional metered-dose sprays (25–200 μL per actuation) predominantly deposit drugs in the anterior nasal cavity [129], limiting OE access and increasing the risk of swallowing, pulmonary inhalation, or unintended systemic absorption. Such limitations have contributed to clinical failures, exemplified by the withdrawal of inhaled insulin formulations due to pulmonary adverse effects and hypoglycemia [130]. Advanced devices integrate formulation properties with device-specific parameters, including particle or droplet size, plume geometry, spray dynamics, nozzle orientation, and airflow, to improve OE deposition.

To date, no delivery device has been shown to selectively target a single intranasal transport pathway. However, optimized devices have achieved up to ~ 45% olfactory deposition in humans [131], with narrow plume angles favoring posterior targeting and mucoadhesive formulations significantly enhancing mucosal retention. Deposition efficiency is strongly size-dependent: tiny particles increase pulmonary exposure, whereas larger particles preferentially impact the nasal vestibule.

Liquid delivery systems, including sprays or electrosprays, generate droplets ranging from nanometers to tens-of-micrometer scales [95]. In contrast, Nebulizers based on vibrating mesh, liquid jet, or ultrasonic mechanisms produce aerosols with diameters of 1–5 µm, resulting in broader nasal dissemination. To maximize olfactory targeting and minimize lung deposition, particle sizes are generally optimized within an intermediate micrometer range (8–12 μm), taking into account spray dynamics and kinetic energy [132]. Dry powder and advanced bidirectional devices further enhance region-specific delivery by directing particles toward the upper nasal cavity and reducing pulmonary exposure. Comparative studies indicate that shorter nozzles of intranasal devices primarily deposit formulations in the anterior nasal cavity, which may favor local therapeutic action but are associated with post administration dripping; while longer nozzles direct the formulations to sphenoid and ethmoid regions [133]. Narrow plume angle furthers assists in deposition to the olfactory region [134]. Further mucoadhesive formulations can increase the residence time up to 14 min [135]. Collectively, these advances have shifted intranasal delivery from nonspecific deposition toward precision, region-targeted platforms optimized for efficient N2B transport.

**Fig. 5:**
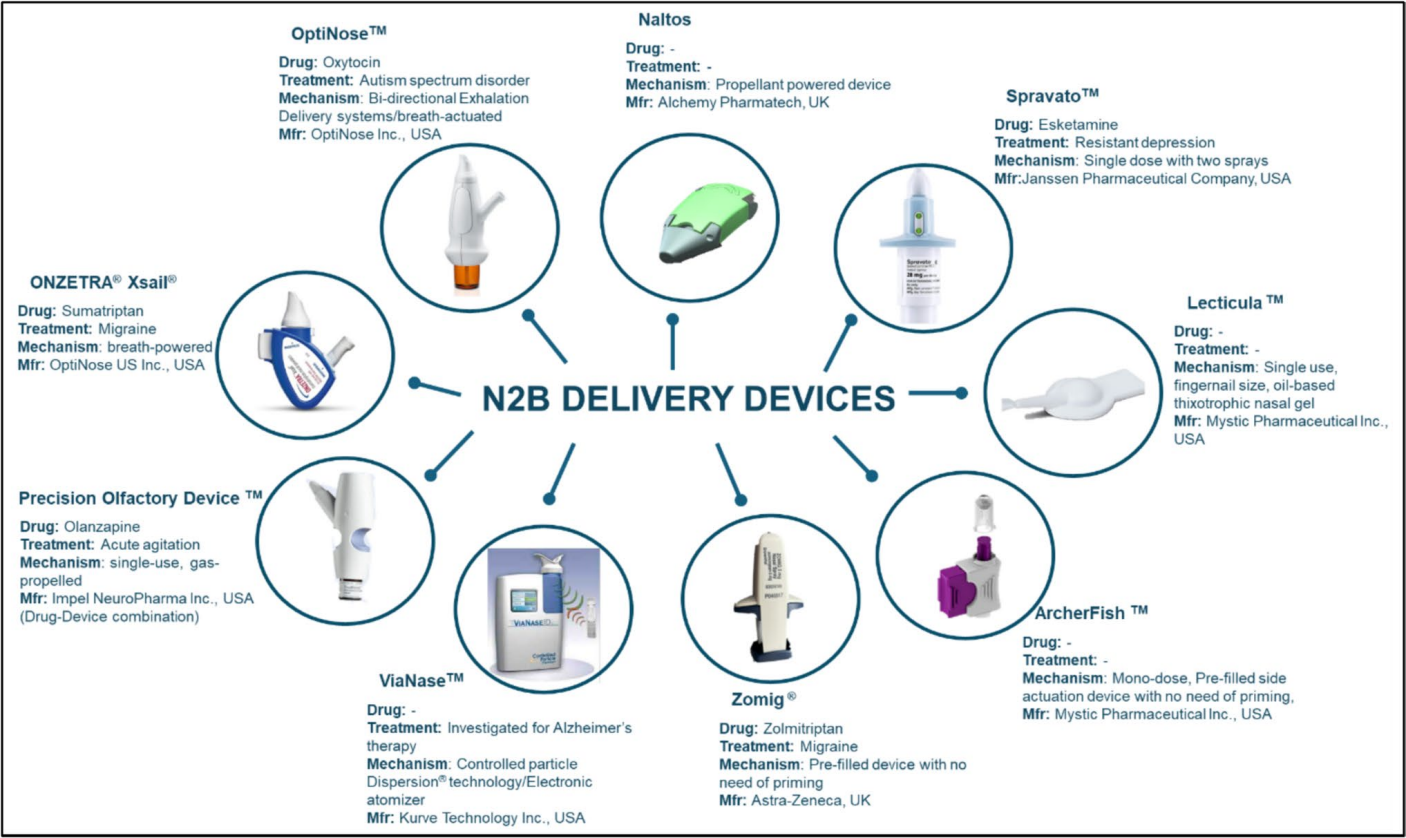
Nose-to-brain delivery using special delivery devices over the years. Modified from [136]

Dry powder intranasal devices typically employ compressed air or an inert gas for particle propulsion, while advanced bidirectional systems utilize patient exhalation to elevate the soft palate and direct aerosols toward the upper nasal cavity, thereby minimizing pulmonary exposure. Recent device innovations have enabled region-specific targeting of the olfactory and upper respiratory regions. More comprehensive reviews on specialized N2B delivery devices can be found in [96, 136–140] and are summarized in Fig. 5.

### Administration techniques

Administration technique is a critical determinant of OE drug deposition. Parameters such as head positioning, breathing pattern, dosing angle, and instillation volume directly influence intranasal distribution [141]. Preclinical intranasal dosing commonly employs micropipettes [142], sometimes fitted with polyethylene tubing to facilitate access to the OE. In rodents, the standard insertion depths are approximately 3 mm in mice and 5 mm in rats [143], with dosing volumes restricted to approximately 5 µL per nostril in mice and 50 µL in rats [143]. Some studies report minimal differences in the positioning of rodents between supine and upright postures during intranasal administration.

In humans, control of head and body movement is necessary to prevent formulation runoff into the nasopharynx or leakage from the nostrils [144]. Inaccurate administration may lead to unintended deposition in non-olfactory regions. Advanced approaches such as ultrasound-mediated delivery, catheter-based administration, specialized intranasal devices, and electrically guided charged particles have been proposed for regional specificity [142].

### Pre-treatment and permeation enhancers

Vasoconstrictors, such as phenylephrine, reduce nasal mucosal blood flow, limiting systemic uptake via venous or lymphatic routes and thereby enhancing drug accumulation in OE [111, 145]. For example, co-administration of 1% PHE significantly increased OE retention and olfactory bulb levels of neuropeptides, such as hypocretin-1 and d-KTP, while reducing systemic and trigeminal distribution by up to 65%, illustrating selective targeting of neural pathways [146]. However, the efficacy of vasoconstrictors may be compound-specific, as long-acting sympathomimetics, such as ephedrine, have not consistently enhanced OE-mediated delivery [147]. Bile salts and its derivatives [148, 149], surfactants [149, 150], fatty acids [151], chelators [152], and other excipients, transiently modulate tight junctions and membrane fluidity to facilitate absorption of polar compounds, peptides, and high-molecular-weight drugs [145, 153]. While effective, the specific impact of their use on OE-targeted delivery remains underexplored.

Cell-penetrating peptides (CPPs) also provide additional mechanisms for intracellular transport. CPPs, such as penetratin, TAT, polyarginines, transportan, lactoferrin, and melittin, traverse cell membranes without causing significant damage, facilitating the delivery of hydrophilic or poorly permeable molecules [154, 155].

Although the enzymatic environment in the nasal cavity is less aggressive than that in the gastrointestinal tract, it can still pose a barrier to N2B delivery [145]. As discussed in "Physiological barriers – mucus and mucociliary clearance", OE contains a range of metabolizing enzymes for xenobiotics as well as odorant molecules to prevent receptor oversaturation [156]. To mitigate enzymatic degradation and enhance drug stability and bioavailability, the use of enzymatic inhibitors has been investigated. Compounds such as bestatin, amastatin, boroleucine, fusidic acids, aprotinin, Cyclosporine A, Camostat mesylate, and phospholipids have demonstrated potential to preserve therapeutics in the nasal cavity, thereby improving local retention and efficacy [8, 156].

OE also possesses tight junctions; however, their manipulation carries the risk of irreversible epithelial damage. A vasodilator, papaverine, was studied for its effect on vascular permeability and tight junction integrity [157]. Papaverine reduced the levels of phosphorylated-occludin—tight junction proteins, thereby increasing the amount of gemcitabine reaching the brain.

## Nanoparticle formulations

Formulation design directly influences drug stability, nasal residence time, epithelial permeability, regional targeting, and CNS bioavailability. A wide range of formulation platforms has been investigated for N2B delivery, including solutions and sprays, liposomes, microemulsions, nanoemulsions, nanoparticles, dry powders, and in situ gelling systems [36, 75, 86, 124, 137, 140, 158–163]. These formulations are often used in conjunction with specialized delivery devices to optimize the deposition within OE.

### Types of nanoparticles 

Nanoparticles (1–100 nm) offer versatile platforms for OE-targeted delivery by improving local enrichment, selectively interacting with specific cell populations, controlling release, and facilitating neuronal uptake while protecting encapsulated drugs from enzymatic degradation [162, 164–166]. Additionally, nanoparticle encapsulation can transiently mask unfavorable physicochemical properties of drug molecules, thereby improving their apparent stability, permeability, and therapeutic efficacy. Both organic (lipid-based, polymeric) and inorganic nanoparticles have been investigated, with surface functionalization enabling receptor-mediated targeting via transferrin, lactoferrin, or insulin receptors expressed on the OE or BBB. Biologically inspired carriers, including viral vectors, exosomes, and biomimetic nanoparticles, facilitate intracellular transport and neuronal uptake while minimizing immunogenicity.

Polymeric nanoparticles formulated using materials such as poly(lactic-co-glycolic acid) (PLGA), chitosan, and PEGylated polymers have also shown promise for N2B delivery. Notably, polymeric nanoparticles have been reported to preferentially utilize the trigeminal nerve pathway for brain transport [167], suggesting that formulation properties may influence the dominant route of CNS entry.

Unilamellar anionic liposomes (~ 100 nm) containing donepezil hydrochloride prepared from hydrogenated soy phosphatidyl choline and cholesterol and dispersed in a gellan gum matrix achieved nearly a four-fold increase in brain maximum concentration compared to oral administration, with peak levels observed within 30 min [168]. Similarly, PEGylated DSPE liposomal encapsulating H102 (112.2 nm, −2.96 mV) for Alzheimer’s therapy protected the peptide from degradation and sustained release, with 1% chitosan further increasing hippocampal accumulation 2.9-fold without detectable nasal toxicity [169]. Due to the extensive literature available on nanotherapeutics [32, 87, 94, 97, 116–120, 127], the authors have limited their detailed discussion on this topic to the present review.

Biologically inspired carriers, including biomimetic nanoparticles such as viral vectors and exosomes, facilitate intracellular transport and neuronal uptake while minimizing immunogenicity. They have widely explored N2B delivery in CNS-related disorders [137, 170–173], although their translational feasibility and long-term safety remain under active investigation. Bioinspired systems, including engineered *Lactobacillus plantarum WCFS1*, have also been explored for localized delivery of peptides and neuroactive hormones, exploiting natural host–microbe interactions to improve nose-to-brain transport [174].

### Surface modification for muco-penetration, adhesion, and receptor targeting

**Fig. 6:**
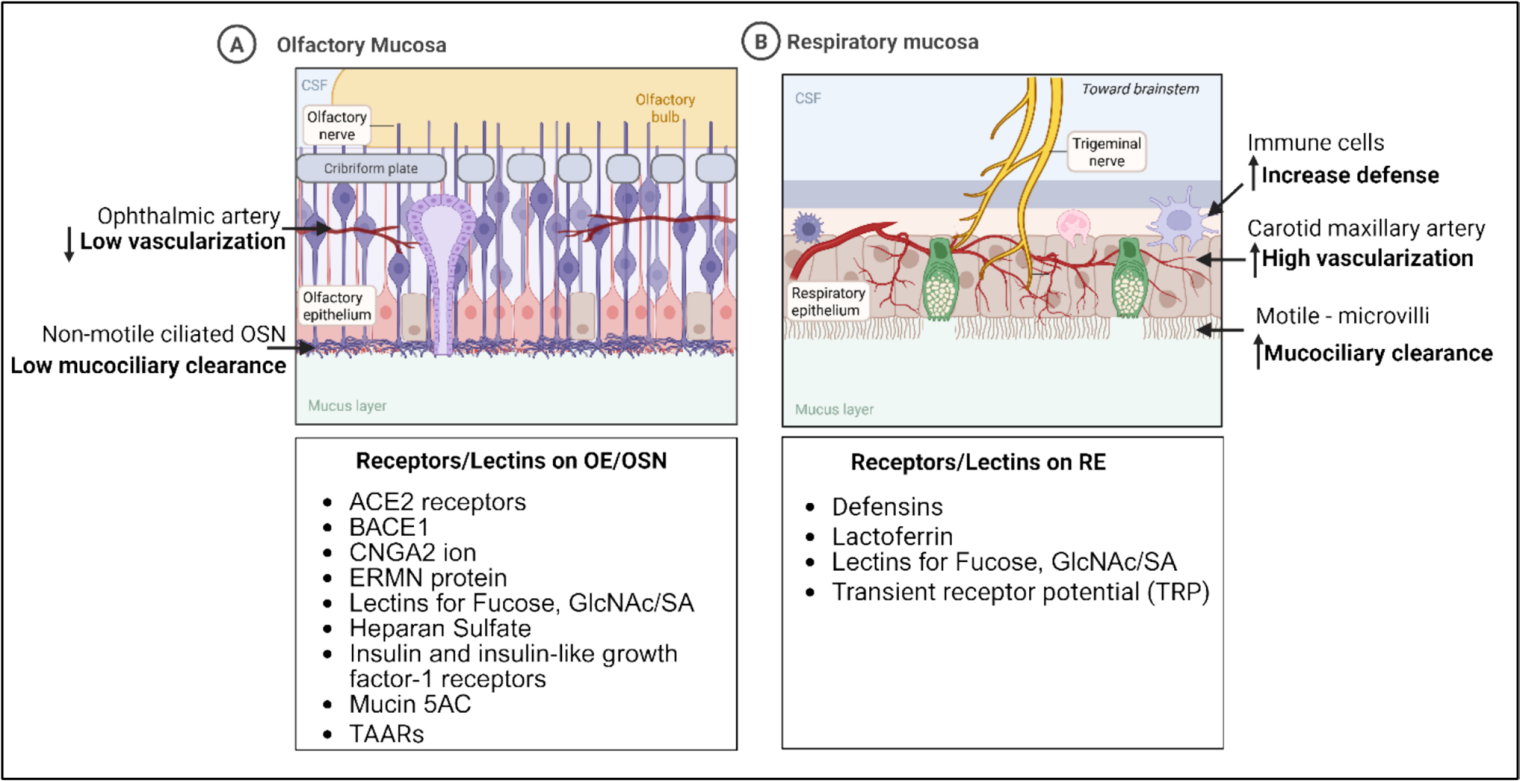
Molecular and physiological variations between (**A**) olfactory epithelium (OE) and (**B**) respiratory epithelium (RE), relevant to targeted delivery and for formulation considerations. Created in BioRender https://BioRender.com/h64cpyr and MS PowerPoint. Angiotensin-converting enzyme-2 (ACE2), fucose, insulin and insulin-like growth factor-1 receptors, N-acetyl galactosamine (GalNAc) and Sialic acid (SA), Trace Amine receptors (TARs), Trace amine-associated receptors (TAARs), and β-Site Amyloid Precursor Protein (APP)-cleaving Enzyme 1 (BACE1)

There are histological, cellular, immunological milieu, receptor, vascularity, enzymatic, glycoconjugate, and molecular marker differences between the OE and RE of the nasal cavity (Fig. 6) [29]. Surface functionalization of nanoparticles represents a versatile strategy to enhance OE targeting by improving mucoadhesion, receptor-mediated uptake, and intracellular transport. Charge-based modifications, such as cationic coatings, increased electrostatic interactions with negatively charged mucin and epithelial surfaces, promoting prolonged residence within the nasal cavity and facilitating local uptake as discussed in "Formulation approaches".

Lectin- and receptor-mediated targeting exploits specific interactions with surface molecules on the OE. Lectins, including *Ulex europaeus agglutinin* I (UEA I) [175] and odorranalectin [176], as well as viral subunits such as cholera toxin B [177], have been used to selectively bind olfactory receptors, enhancing localized delivery and reducing reliance on alternative pathways such as the trigeminal nerve.

Cell-penetrating peptides (CPPs), when combined with nanoparticulate or hydrogel carriers, enhance cellular uptake, tissue penetration, and brain targeting of hydrophilic or poorly permeable molecules [154, 155]. L-penetratin facilitated intranasal delivery of peptides, including insulin and exendin-4, predominantly through the olfactory pathway and circumvented the trigeminal nerve-associated delivery routes [155].

Nanoparticles functionalized with receptor-specific ligands further improve selective uptake and intracellular internalization. OE expresses many differential OE receptors distinct from RE – Trace amine-associated receptors (TAARs), Trace Amine receptors (TARs), Angiotensin-converting enzyme-2 (ACE2), insulin and insulin-like growth factor-1 receptors, β-Site Amyloid Precursor Protein (APP)-cleaving Enzyme 1 (BACE1), fucose, N-acetyl galactosamine (GalNAc) and Sialic acid (SA) expression, which can be explored for possible targeting OE or OSN transport (Fig. 6) [29]. Other receptors, including transferrin receptors, low-density receptor or LDLR-related protein 1, integrin receptors, and epidermal growth factor receptors, have been explored for intracellular and transcellular uptake across epithelial and endothelial cells (Fig. 4E) [49]. However, it remains unclear whether these pathways enable selective or region-specific uptake within the OE, RE, or across the nasal epithelial layer, warranting further investigation.

Strategies that slow mucociliary clearance can further prolong residence time, but for drugs with inherently low permeability, absorption enhancers remain critical to achieving therapeutic CNS concentrations [110, 118]. Nanoparticles may be administered alone or incorporated into secondary carriers, such as gels, dry powders, or in situ gelling systems, to enhance residence time, enhance nanoparticle-mediated transport, and reduce mucociliary clearance [111, 178].

### Particle size, shape, and surface charge considerations

Particle size is critical for OE access. Particles larger than approximately 100 nm are unlikely to undergo intraneuronal transport, as their dimensions exceed the diameter of axons of *Filia olfactoria*, which consist of bundles of olfactory neurons ensheathed by Schwann cells and characterized by narrow paracellular spaces of approximately 10–15 nm [179, 180]. Sub-100 nm nanoparticles can traverse the olfactory epithelium via transcellular or paracellular pathways. In contrast, larger particles (e.g., > 200 nm) show limited penetration [179]. Polystyrene-coated chitosan nanoparticles, sized 200 nm, were not accessible to the olfactory epithelium compared to 80-nm-sized [143]. Nevertheless, neither particle was seen in the olfactory bulbs. Additionally, the diameter of the olfactory sensory neuron ranges from 100 to 700 nm, which may restrict the transportation of particles greater than this [181]. Likewise, nanoemulsions of 900 nm in size exhibited minimal brain delivery, whereas nanoemulsions of 108 nm achieved 4.4% brain accumulation, highlighting the size-dependent transport [182].

Surface charge also influences mucosal diffusion and selection of transport pathways. Cationic particles adhere strongly to negatively charged mucin, glycans, or mucus, enhancing retention. [143, 168]. Anionic or neutral hydrophilic nanoparticles demonstrate improved OE penetration via the olfactory nerve pathway. Cationic [^3^H]-labeled DSPE liposomes (~ 80 nm) have been reported to have a higher accumulation in the anterior parts of the brain, especially the olfactory and forebrain, with less widespread distribution. In contrast anionic liposomes (~ 86 nm) tend to deposit in the bulbospinal tract and hindbrain, with PEGylation further enhancing this distribution pattern [183]. However, some theories also suggest that strong cationic interactions with mucin cause accumulation of particles in the mucosa, promoting the systemic pathway instead of the epithelial pathway [184]. Cations may also retard translocation and follow the trigeminal nerve pathway. Additionally, cationic polymeric nanoparticles transfer to brain parenchyma is slower than that of anionic polymeric nanoparticles due to intra- or extraneuronal pathways [184].

PEGylated liposomes or hydrophilic surfaces favor aqueous paracellular diffusion, whereas hydrophobic surfaces support transcellular transport [166]. The distribution of PEGylated and non-PEGylated, however, remained the same in the trigeminal nerves.

## Clinical translation

N2B has promising potential for brain delivery; however, it must overcome translational limitations.

### Safety and toxicity in the nasal cavity

N2B delivery offers a significant advantage of bypassing the BBB; however, its clinical translation requires rigorous evaluation of safety, tolerability, and regulatory compliance [111]. Because N2B formulations are in direct contact with the nasal mucosa and OE, preservation of epithelial integrity, OSN, and supporting tissues is critical. Structural or functional damage to these structures may result in impaired olfaction, cognitive dysfunction, or long-term neurotoxicity, rendering local and central safety assessment a regulatory prerequisite.

In addition to anatomical constraints, successful clinical translation also depends on rigorous optimization of formulation composition [143]. Active compounds must demonstrate compatibility with nasal tissues, including minimal irritancy and predictable absorption behavior. Physicochemical properties of drugs must be carefully considered. CNS-acting drugs may not always be suitable candidates for nasal delivery; for example, irritant molecules may require encapsulation within protective carriers to minimize mucosal damage [185]. Regardless of dosage form (solution or powder), the drug must rapidly dissolve in nasal secretions, permeate the mucosa efficiently [110], and if it is a carrier-based, traverse the epithelium either in dissolved form or intact and undergo predictable biodegradation into non-toxic metabolites.

Formulation excipients incorporated to enhance stability, solubility, or bioavailability, including permeation enhancers, mucoadhesive polymers, and targeting ligands, must demonstrate biocompatibility with nasal tissues [186]. Nasal mucosa may not have the same tolerability as the drugs [187]. Certain penetration enhancers and surfactants may elicit immune or inflammatory responses, requiring a balance between efficacy and mucosal safety [188]. Antimicrobials and preservatives used for stability exhibit ciliotoxicity – decreased cilia beat frequency or ciliostatic effect, and decreased rate of nasal clearance [187]; while some cholinergic agents stimulate ciliary activity. They should also be assessed for their effect on mucociliary clearance, ciliary beat frequency, and recovery of damaged mucosa, not only for short-term intranasal administration but also for their impact on long-term and repeat administration outcomes, particularly under different pathological conditions.

Regulatory agencies favor GRAS-designated excipients used within acceptable concentration limits. Key formulation parameters include pH (5.0–6.5), viscosity, and osmolarity [44, 146]. Hypotonic formulations have been associated with increased epithelial injury, whereas isotonic or mildly hypertonic systems tend to preserve mucosal integrity [189]. Moreover, hypertonic formulations with 1700 mOsm/l or more increase the mucociliary clearance [190]. Although the effects of osmolarity on drug bioavailability remain inconclusive, an osmolarity range of 200–600 mOsm/L is widely recommended to maintain mucosal health and patient comfort [95]. Buffer selection is also relevant, as acetate buffers are typically more irritating than citrate, phosphate, or adipate buffers [189].

Nanotechnology-based N2B formulations introduce additional regulatory complexity, as nanoscale size can alter the physicochemical, biological, and toxicological properties of drugs [191]. Enhanced cellular uptake associated with submicron particles may increase the risk of inflammation, cellular stress, and altered cell function, particularly for non-biodegradable materials. Therefore, biodegradable carriers, surface modification strategies, and thorough long-term safety evaluation are strongly recommended [162, 192]. The drug-loading capacity of nanoparticles is a critical determinant of the achievable dose and may directly influence the required dosing frequency [54]. Furthermore, stabilizers incorporated to maintain nanoparticle integrity during manufacturing and storage may influence long-term safety profiles and must be thoroughly evaluated [91].

Safety assessment should account for physiological and pathological nasal conditions, environmental variability, and administration technique, as deposition site influences both efficacy and off-target exposure. Formulations must maintain stability, potency, and reproducibility across these variables. Delivery devices may improve targeting and reduce nonspecific distribution. Anterior deposition toward the RE may increase systemic exposure, whereas deposition toward the nasal floor may enhance mucociliary clearance [193]. Appropriately designed delivery devices can improve targeting specificity and minimize off-target exposure [111].

Comprehensive in vitro and in vivo investigations are essential to evaluate cytotoxicity, inflammatory responses, nanoparticle-related toxicity, and structural integrity of nasal tissues [191].

Commonly employed models for in vitro toxicity include:

- **Local tolerability assessments** incorporating nasal humidity, temperature, humidity, or disease conditions such as rhinitis.
- **Cytotoxicity testing** on primary or immortalized cell lines of nasal mucosa collected from the olfactory region [194] or an immortalized RPMI 2650 cell line isolated from squamous cell carcinoma of the nasal septum (comprehensive model of RE and OE) [195]
- **Neurotoxicity evaluation** on neuronal cells/olfactory nerve to determine safety using LDH or MTT assays using conventional cell lines in Transwell or microfluidics.
- **Irritation and tissue damage studies** using the erythrocyte lysis model or histology, ciliary function using chicken embryo tracheal tissue [145]
- **Permeation studies,** including transepithelial electrical resistance measurements [196] are useful for evaluating epithelial barrier integrity. This may be important in some contexts like infection, where permeability may be altered.
- **Ciliotoxicity** using mucociliary activity or ciliary beat frequency (PS: OE does not show beating motion [70], ciliary moment, hemorrhage, or necrosis on fresh nasal mucosa of pig, sheep, or suitable animals [188]
- **BBB penetration studies** using Transwell or microfluidic BBB models [197]
- **Computational modeling** to predict CNS exposure and potential side effects [198]
- **In vivo neurobehavioral assessments** e.g., functional observation battery or modified Irwin screen [199], to detect neurotoxicity in brain and nerve tissue
- **Evaluation of Olfactory and cognitive functions**, particularly dependent on the olfactory bulb and prefrontal cortex [200, 201].
- **Assessment of sub-toxic irritation**, e.g., measuring evoked potential/electrophysiological measurement or immunocytochemical markers, e.g., c-Fos protein, could be a prelude for human subject tests. Alternatively, irritation models such as lactate dehydrogenase released from the foot mucosa of the slug have also been proposed [145, 202].
- **Histopathological examination** of the nasal, brain, and neural tissue.
- **Immunological function:** The microglia in the olfactory bulb has heightened immune sensitivity, as they can be easily activated compared to other brain regions. Hence, immunological activity of olfactory Ensheathing cells and microglial cells of olfactory bulbs should be considered [83] when treated with formulations.

It is important to recognize that certain in vitro models may overestimate toxicity [203]; therefore, results should be interpreted within an integrated, weight-of-evidence risk-assessment framework [204]. Finally, non-invasive imaging approaches such as olfacto-scintigraphy using Thallium-201 offer valuable tools to assess olfactory nerve integrity and tracer migration to the olfactory bulb, supporting both safety evaluation and clinical translation [144].

### Clinical trials of N2B formulations

Encouraging preclinical evidence has driven the translation of N2B delivery strategies into a growing number of clinical investigations evaluating CNS efficacy [91, 110, 205]. However, direct extrapolation of rodent data to humans is limited by interspecies differences [206]. When normalized to body weight (Fig. 1), the OE surface area in humans is markedly smaller than in rodents. The maximum intranasal volumes that can be administered also differ across species, ranging from 5—50 μL in rodents [27] and 100–150 μL in humans [86]. Additional anatomical and physiological differences between species are non-linear, further reducing deposition efficiency and limiting the predictability of clinical outcomes [11, 44]. Reliable scaling factors for CNS exposure across species remain undefined [35, 207].

To date, 150 clinical trials are under investigation. Table 1 shows some of the approved N2B formulations. However, the studies may not differentiate between the uptake of drugs through OE, RE, or systemic circulation. The selective targeting of a single intranasal transport pathway has not yet been explicitly evaluated. Moreover, none of the nanoformulations have achieved clinical translation so far.

**Table 1:** Some of the approved N2B drugs targeting CNS diseases from 2019 onward [91, 110, 205]

Drugs	Treatment Condition	Year of study	Clinical trial	Nasal Device	Key outcomes	Sponsor/Manufacturer
Esketamine Hydrochloride	Treatment-resistant depression	2019	Approved	Disposable pre-filled Nasal spray device	Abolute bioavailabity is ~ 48%, while it 8% via oral administration [208]	Spravato® (Janssen Pharmaceuticals)
Midazolam	Epilepsy	2019	Approved	Disposable pre-filled Nasal spray device	A 5 mg dose terminated Seizure within 10 min and remained seizeuree free up to siz hours [209]	Nayzilam® (Ucb Inc.)
Sumatriptan	Migraine	2019	Approved	Disposable pre-filled Nasal spray device	Onset of relief begain within 15 min. 20 mg dose was more effective than 10-mg dose [210]	Tosymra® (Upsher Smith Laboratories)
Diazepam (NRL-1)	cluster or acute repetitive epilepsy	2020	Approved	Disposable pre-filled Nasal spray device	unique combination of a vitamin E-based solution with Intravail®	Valtoco® (Neurelis)
Varenicline	Dry eye Disease	2021	Approved	Reusable nasal spray device	stimulates nicotinic cholinergic receptors and basal tear film production through the trigeminal parasympathetic nerve pathway [211]	Tyrvaya (Oyster Point)
Dihydroergotamine Mesylate	Migraine	2021	Approved	Precision Olfactory Delivery (POD)	rapid onset of freedom from pain from single use- 36.6% within 2 h, 67.1% within 4, and 85.5% withing 24 h post dose of treated attacks [212]	Trudhesa® (Impel)
Naloxone Hydrochloride	Opioid overdose	2021	Approved	Disposable pre-filled Nasal spray device	8 mg of naloxone, 41.6%−47.6% bioavailable [213]	Kloxxado® (Hikma)
Zavegepant	Migraine	2023	Approved	Disposable pre-filled Nasal spray device	The 10 mg nasal spray had high efficiency from pain at 2 h lowest adverse side effects but similar to oral calcitonin gene-related peptide receptor antagonists [214]	Zavzpret® (Pfizer)
Naloxone Hydrochloride	Opioid overdose	2023	Approved	Disposable pre-filled Nasal spray device	4 mg of naloxone, multiple naloxone administrations may be needed per overdose event [215]	Narcan, (Amphastar Pharmaceuticals)

### Regulatory guidelines and challenges

Regulatory harmonization for nasal formulations targeting the brain is still incomplete. While well-established frameworks exist for nasal and inhalation products, specific guidance for delivery via the olfactory or trigeminal pathways is still evolving. This regulatory uncertainty contributes to extended development timelines and increased strategic complexity.

Most N2B delivery systems are regulated as drug-device combination products, requiring an integrated evaluation of the drug substance, formulation, device performance, and user interaction. Regulatory agencies mandate the demonstration of safety, efficacy, quality, and stability through a risk-based approach that encompasses nonclinical, clinical, and human factors studies. Compliance with combination-product regulations ensures consistent dose delivery, functional reliability, and patient safety throughout the product lifecycle.

The U.S. Food and Drug Administration’s June 2024 draft guidance, “Essential Drug Delivery Outputs for Devices Intended to Deliver Drugs and Biological Products,” defines Essential Drug Delivery Outputs (EDDOs) as critical performance attributes necessary to maintain intended drug delivery [216]. For nasal spray combination products, EDDOs include delivered dose (spray weight), content uniformity, spray pattern, plume geometry, and droplet or particle size distribution, as well as actuation-related parameters such as activation force. For inhalation-based combination products, including metered-dose inhalers and nebulizers, relevant EDDOs may additionally include aerodynamic particle size distribution, priming and repriming behavior, dose counters and their accuracy, device actuation force and counter-actuation, breath synchronization, patient interface performance, and delivery rate, depending on the device design.

Regulatory review also extends beyond device performance to include drug-device compatibility, sterility assurance, biocompatibility, human factors, extractables and leachables, and electrical safety and radio frequency wireless technology or electromagnetic safety, where applicable. Chemistry, manufacturing, and controls (CMC) requirements, in vitro performance testing and labeling expectations are detailed in FDA guidance for Nasal Spray and Inhalation products [217]. United States Pharmacopeia (USP) chapters addressing aerosols, sprays, powders, USP < 601 >, and nebulizers, USP < 1601 >, and corresponding European Medicines Agency guidance on pharmaceutical quality of inhalation and nasal medicinal products [218, 219].

For locally acting nasal formulations, regulatory approval typically relies on in vitro bioavailability and bioequivalence testing, including single-actuation content through container life, drug content, particle and droplet size, droplet distribution by cascade impactor, spray pattern, priming/repriming performance, and plume geometry [220]. In certain cases, particularly with suspension formulations, pharmacokinetic studies may be required to characterize systemic exposure and minimize its systemic effects.

Excipients and additives within combination products must be pharmacologically inert, non-irritating, and suitable for chronic administration, as repeated exposure may affect both tissue compatibility and device performance [145]. Additionally, human factors engineering and usability testing are critical regulatory components, ensuring safety and its effective use by the intended patient population under real-world conditions. Less-friendly devices may lead to uncomfortable administration, discomfort, or inconsistent dosing, thereby compromising therapeutic outcomes.

Nevertheless, consistent delivery to OE and reproducible brain exposure have not yet been demonstrated across patient use or devices. Moreover, the conventional bioavailability and bioequivalence evaluation of all nasal products does not yet speak about CNS exposure or clinical efficacy. Also, human factors engineering is the most critical challenge and requires extensive testing and optimization.

## Future directions

Several challenges for N2B highlight the importance of advanced modeling approaches and human-relevant experimental systems in the development of clinically translatable delivery platforms [221].

### Personalized delivery systems

Recent advances in three-dimensional (3D) printing and medical imaging have enabled the fabrication of patient-specific nasal cavity replicas derived from computed tomography scans, offering new opportunities for personalized N2B delivery. These anatomically accurate nasal casts provide physical models that closely replicate individual nasal geometries and are increasingly used to optimize formulation attributes, device design, and administration parameters to improve regional drug deposition, particularly within the OE [131].

Unlike standardized nasal casts that represent averaged population features, personalized 3D-printed replica models capture interindividual anatomical variability, which is critical given the substantial differences in nasal morphology across age, sex, and ethnic groups. Although these replicas lack the biological properties of nasal mucosa**,** most notably mucoadhesion**,** they can be modified with artificial mucus coatings to better approximate in vivo conditions [95, 222]. Such models are valuable for evaluating spray penetration patterns, vertical and regional distribution, and quantifying deposition using analytical or colorimetric techniques. However, their predictive utility is confined to device-to-target transport and deposition and does not fully account for subsequent processes such as diffusion, permeation, or brain accumulation.

Several studies have demonstrated the application of personalized nasal casts to investigate OE targeting under varying anatomical and operational conditions. For example, dry powder formulations have been assessed for OE deposition in male and female nasal geometries, including models with septal perforations, using both unidirectional and bidirectional delivery approaches in the presence of artificial mucus [222, 223]. Key variables such as administration angles, nostril selection, inspiratory flow, and device design were shown to significantly influence deposition within the olfactory cleft and internal nasal valve regions. Such approaches may inform individualized optimization of N2B delivery strategies.

Concurrently, patient-specific nasal delivery devices are emerging as a complementary strategy. Menegatou et al. developed a personalized matrix–piston nasal device fabricated using 3D printing with thermoplastic polyurethane and acrylonitrile butadiene styrene, designed to position polymeric drug-loaded films directly onto the OE region [224]. While promising, these systems currently require further validation in human subjects and are limited by small drug-loading capacities (approximately 25 μL) to form thin film formulations.

Industrial efforts further underscore the momentum toward personalization. Aptar Pharma has introduced the “Aeronose” nasal cast platform to support in vitro evaluation of OE-targeted delivery systems [225, 226]. Similarly, Nemera (IL, USA) has developed specialized nasal spray configurations for insulin delivery, achieving over 50% deposition in the olfactory cleft in human studies. To enhance usability and reproducibility, wearable sensor-guided headsets have been engineered to help patients maintain optimal device orientation during administration. Nemera’s Guided Stream™ technology, based on human factors engineering and voice of the patient and patient-centered design principles, helped to minimize irritation and improve comfort, is anticipated to undergo further evaluation in 2025 [227, 228].

Collectively, these emerging technologies underscore the increasing importance of personalized anatomical models and device configurations in advancing precision N2B drug delivery.

### Smart and stimuli-responsive delivery systems

Smart drug delivery systems exploit materials that respond dynamically to local physiological and pathological cues, enabling controlled and site-specific bioresponsive drug release. In brain-targeted applications, 3D crosslinked hydrogels are of particular interest as they partially recapitulate the brain extracellular matrix and respond to stimuli such as temperature, pH, ionic strength, and reactive oxygen species (ROS) [154].

In situ thermos-responsive mucoadhesive gels have been studied for the intranasal delivery of anti-Parkinsonian drugs such as selegiline hydrochloride [229, 230] ropinirole [231], and for anti-Alzheimer’s drugs including tacrine [232]. These drugs are limited by low brain bioavailability, poor oral absorption, and extensive hepatic metabolism [233], while ropinirole additionally exhibits a short biological half-life [234]. Incorporation into thermoresponsive gels has been shown to preserve gel integrity after administration, typically producing an initial burst release followed by sustained drug release for up to 8 h, resulting in improved brain bioavailability compared with conventional drug solutions. Elevated ROS levels associated with neurodegenerative disorders have been leveraged using ROS-responsive olanzapine-loaded nanoparticles incorporated into thermoresponsive poloxamer (Pluronic®) hydrogels [235]. Following intranasal administration, the formulation gels within the nasal cavity, allowing for sustained drug release and enhanced brain exposure. Similar approaches using curcumin incorporated into thermoresponsive gels have demonstrated distribution to multiple brain regions, primarily via the olfactory and trigeminal pathways, with a secondary contribution from systemic absorption [236].

Ion- and pH-responsive polymeric systems further expand the repertoire of smart nasal delivery platforms. Chitosan-based formulations remain soluble under acidic conditions but undergo gelation to form viscoelastic colloids as the pH increases to physiological nasal values (approximately pH 6.2–6.8), thereby enhancing mucoadhesion and retention [237]. Similarly, polyacrylic acid derivatives such as Carbopol exhibit pH-dependent swelling and gel formation [154]. In contrast, polysaccharides such as gellan gum undergo sol–gel transitions in the presence of mono- or divalent cations, where ionic crosslinking induces conformational rearrangement and gel formation, providing another mechanism for environment-triggered drug release [238, 239].

Collectively, these smart and stimuli-responsive platforms offer multifunctional delivery platforms that integrate environmental sensing, prolonged residence, and enhanced tissue penetration. Although most remain at the investigational stage, their integration with disease-specific triggers and personalized or digitally enabled platforms holds significant promise for next-generation precision therapies targeting central nervous system disorders.

### Integration with digital health

The integration of advanced drug delivery systems with digital health technologies, artificial intelligence, and machine learning is accelerating the transition toward digitally enabled therapeutics. While smart technologies are well-established for intranasal delivery to address local and respiratory conditions, such as the common cold, pain, and rhinitis, these technologies are readily extendable to N2B delivery for CNS disorders. Growing industry engagement reflects increasing interest in embedding digital functionality within intranasal delivery platforms [240, 241]. Importantly, these innovations operate within an integrated digital ecosystem that encompasses patient monitoring, device optimization, and regulatory and safety oversight.

#### Patient centered digital platforms

Patient-centered digital platforms enable longitudinal screening, diagnosis, and monitoring using multimodal data streams, including speech analysis, gait patterns, eye tracking, cognitive testing, and wearable-derived physiological metrics. Integration with mobile health applications and wireless connectivity, these tools support real-time adherence monitoring, dosing reminders, remote consultations, and data-driven personalization of therapy with immediate evaluation. Such approaches have shown promise in neurodegenerative disorders, including Alzheimer’s disease and dementia, where continuous monitoring of cognitive and behavioral endpoints can inform treatment decisions [242, 243].

#### Device-centered digital platforms

Device-centered digitalization complements these approaches by incorporating sensors and feedback mechanisms directly into drug delivery systems to guide correct nozzle placement, optimize administration angle, and ensure accurate dose delivery to OE during self-administration. These features have the potential to reduce variability in drug deposition, improve therapeutic reproducibility and delivery to the brain, and enhance patient confidence and compliance.

#### Regulatory- and safety-centered digital platforms

At the regulatory and safety level, digitally connected delivery systems facilitate controlled dosing of highly regulated therapeutics and address concerns related to the misuse. For example, FDA has authorized a digital application for opioid use disorder management [244]. Internet-of-Things (IoT)-enabled inhalation platforms restrict drug access via biometric authentication [245]. Such systems enable real-time dose tracking, cloud-based data capture, and immediate transmission of administration data to healthcare providers, thereby supporting controlled use, pharmacovigilance, post-marketing surveillance, and regulatory compliance.

Collectively, the integration of patient-derived data, intelligent device design, and regulatory connectivity with advanced N2B delivery technologies establishes a closed-loop framework for N2B therapeutics, advancing precision, safety, and accountability in CNS drug delivery.

## Conclusion

This review highlights recent advances and emerging strategies in N2B drug delivery, underscoring its growing potential for treating CNS disorders. Although N2B transport is primarily mediated via the olfactory and trigeminal nerve pathways, particular emphasis was placed on OE-targeted delivery as a means to achieve greater site specificity and enhanced brain deposition while bypassing the BBB. The clinical success of several FDA-approved intranasal therapies for neurological conditions such as epilepsy, migraine, and depression further supports the translational promise of this approach.

Despite being non-invasive, effective N2B delivery requires careful consideration of multiple factors, including nasal anatomy, site of drug deposition, physiological and environmental barriers, and inter-patient variability. Numerous physicochemical and formulation-based strategies have been explored to improve delivery efficiency; however, variability in clinical translation persists, revealing a gap between preclinical success and therapeutic implementation. Currently, no technique has been demonstrated to selectively target a specific uptake pathway through the olfactory epithelium. Addressing challenges related to anatomical constraints, dose-volume limitations, and safety remains critical for advancing this field.

Moreover, hurdles associated with industrial scale-up, regulatory pathways, and clinical validation continue to limit the widespread adoption of N2B therapeutics. Nevertheless, ongoing multidisciplinary efforts are expected to alleviate these barriers, particularly for advanced modalities such as gene-based therapies. Future research should focus on achieving precise targeting of specific brain regions or cell types, clarifying the relative contributions of olfactory versus trigeminal pathways, and elucidating the transport mechanisms of nanoparticle-based systems. Such integrated investigations will be essential to fully realize the clinical potential of N2B drug delivery.

## Data Availability

No datasets were generated or analysed during the current study.

## Abbreviations

ACE2: Angiotensin-converting enzyme-2
BACE1: β-Site Amyloid Precursor Protein (APP)-cleaving Enzyme 1
BBB: Blood – Brain-Barrier
CNGA2: Cyclic nucleotide-gated
CNS: Central Nervous System
CSF: Cerebrospinal fluid
DSPE: 1,2-Distearoyl-sn-glycero-3-phosphoethanolamine
GalNAc: N-acetyl galactosamine
N2B: Nose-to-brain
OE: Olfactory Epithelium
RE: Respiratory Epithelium
SA: Sialic acid
TAARs: Trace amine-associated receptors
TARs: Trace Amine receptors
